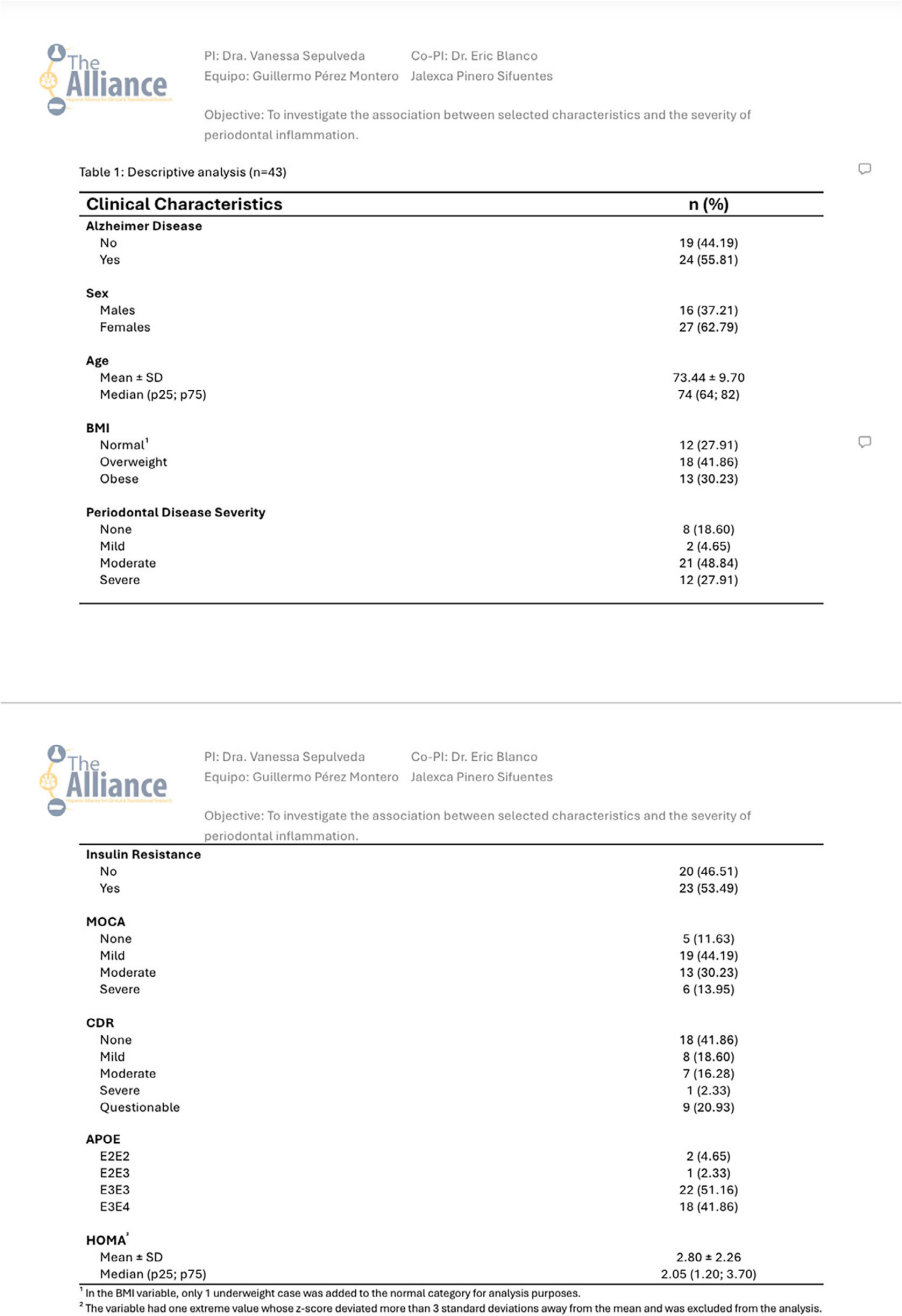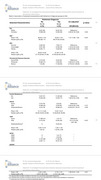# Dementia Severity and Aging as Primary Predictors of Periodontal Inflammation: A Pilot Study in a Puerto Rican Alzheimer’s Cohort

**DOI:** 10.1002/alz70861_108947

**Published:** 2025-12-23

**Authors:** Eric J Blanco, Ramon Gonzalez, Guillermo Perez, Jalexca Piñero, Hiram Morales, Gerianne Olivieri‐Henry, Christian Gonzalez, Filipa Godoy‐Vitorino, Vanessa Sepulveda

**Affiliations:** ^1^ University of Puerto Rico, Medical Sciences Campus, School of Medicine, San Juan, PR USA

## Abstract

**Background:**

Periodontal inflammation has been implicated in Alzheimer’s Disease (AD) through systemic inflammatory and neurodegenerative pathways, including microbial dysbiosis and cytokine signaling and microbial infiltration. While the oral microbiome’s role in cognitive decline has gained momentumrecently, there is limited research on these associations in Hispanic population, underrepresented in Alzheimer’s research and facing disproportionately high burden of both dementia and oral disease.

**Objective:**

To evaluate the association between periodontal disease severity and clinical, cognitive, metabolic, and genetic factors in a cohort of Puerto Rican older adults with and without Alzheimer’s Disease.

**Methods:**

We conducted a cross‐sectional analysis of 43 community‐dwelling participants enrolled in the Association‐Gut‐Microbiome‐AD (IRB: 2290033626) study. Each underwent a full‐mouth periodontal examination, including clinical attachment loss (CAL), probing depth (PD), and bleeding on probing (BOP) at six sites per tooth. Periodontal disease severity was classified using CDC–AAP 2012 case definitions: mild, moderate, or severe periodontitis based on interproximal CAL and PDthresholds. Predictor variables included age, BMI, insulin resistance (HOMA‐IR), APOE‐ε4 status, AD, and cognitive scores (MoCA and CDR). Bivariate analyses and Multinomial logistic regression were used to assess associations.

**Results:**

Eighty‐one percent of participants met criteria for periodontitis, including 28% with severe and 49% with moderate disease. Age was the most consistent predictor of worsening periodontal inflammation (p < 0.01). CDR scores were significantly associated with greater severity in both bivariate (p < 0.01) and unadjusted models (CDR mild vs none: OR = 0.13; 95% CI, 0.02–0.86; *p* = 0.03). Severe periodontitis was more commonly observed in Alzheimer’s, showing a higher odds ratio when compared to controls (OR = 2.75; *p* = 0.44) though not statistically significant. MoCA scores, APOE‐ε4 status, BMI, and HOMA‐IR were not significantly associated with periodontal severity.

**Conclusion:**

Aging and dementia severity (CDR) were the strongest predictors of periodontal inflammation. These findings align with emerging literature linking oral microbial dysbiosis and neuroinflammatory mechanisms in AD. The use of standardized CDC–AAP definitions provided clinical rigor, and the elevated periodontitis prevalence observed in this cohort underscores the need for integrated oral‐systemic care strategies in dementia prevention and management, particularly in underrepresented populations such as Puerto Rico.